# The Expression and Transfer of Valence Associated with Social Conformity

**DOI:** 10.1038/s41598-019-38560-4

**Published:** 2019-02-15

**Authors:** Prachi Mistry, Mimi Liljeholm

**Affiliations:** Department of Cognitive Sciences, University of California, Irvine, USA

## Abstract

Consensus seeking – abandoning one’s own judgment to align with a group majority – is a fundamental feature of human social interaction. Notably, such striving for majority affiliation often occurs in the absence of any apparent economic or social gain, suggesting that achieving consensus might have intrinsic value. Here, using a simple gambling task, in which the decisions of ostensible previous gamblers were indicated below available options on each trial, we examined the affective properties of agreeing with a group majority by assessing the trade-off between social and non-social currencies, and the transfer of social valence to concomitant stimuli. In spite of demonstrating near-perfect knowledge of objective reward probabilities, participant’s choices and evaluative judgments reflected a reliable preference for conformity, consistent with the hypothesized value of social alignment.

## Introduction

Social animals, from honeybees to humans, must often reach consensus with other group members when making collective decisions. By agreeing with a majority opinion, individuals are able to avoid social rejection and retain access to group resources^1,2^. While consensus seeking in the face of conspicuous contingent reward is unsurprising, individuals also consistently conform in the absence of any apparent social or economic gain^3–7^, suggesting that the act of agreeing with a majority might have *intrinsic value* – a notion that is further supported by recent neuroimaging work demonstrating an overlap between neural substrates mediating conformity and those involved in processing reward^4,6,8–10^. Here, we use a simple gambling task to further characterize the valence associated with conformity and dissent.

In spite of ample evidence of apparently inconsequential conformity, it is problematic to conclude that conforming decisions are rewarding simply because such decisions are made. Error-based adjustments towards a reference, such as a majority opinion, need not be associated with hedonic valence but may simply reflect an effort to approximate accuracy by minimizing expectation violations. Moreover, the apparent involvement of brain regions frequently implicated in reward processing does not warrant the reverse inference that conforming decisions have a hedonic component; first, since those same neural regions also respond to valence-neutral but surprising, or otherwise salient, stimuli^11–14^ and, second, because neural signals identified in social conformity studies often appear more consistent with error adjustment than with hedonic reinforcement (see Discussion for details). There is a clear need, thus, for studies that employ independent measures of the valence associated with conformity and dissent.

Some social psychology studies have used evaluative measures to assess emotional constructs associated with dissent from group opinions. For example, Matz and Wood^15^ used an emotion measure to assess dissonance discomfort, negative self-evaluation and positive feelings associated with agreeing or disagreeing with a group of ostensible peers. They found that participants who disagreed with the group experienced significantly greater dissonance discomfort than those who agreed, especially if they believed that they would be required to discuss their opinions or reach consensus with other group members. While no such effects were found for measures of negative self-evaluation and positive feelings, in a subsequent study, positive feelings increased and negative self-evaluation decreased when participants were given the opportunity to achieve consensus by persuading others or joining a more congenial group. This and related work suggests that some form of valence does accompany decisions made relative to a group norm. However, lacking a formal framework of reward-based behavior, the approach is poorly suited to quantify hedonic aspects of social conformity.

Notably, while the phenomenon of apparently inconsequential conformity has been demonstrated across social psychology and neuroscience literatures^3–6^, and while the notion that individuals conform to social norms at considerable personal cost is well rooted in evolutionary and social sciences^16^, no previous study has, to our knowledge, provided direct experimental and formal evidence for a willingness to pay a price in order to conform. In Experiment 1, we directly pit the value of conforming against an alternative incentive, exploring a trade-off between social and non-social currencies. In Experiment 2, using a *conditioned reinforcement* procedure, we further probe the affective valence of majority alignment by assessing the transfer of such valence to concomitant stimuli.

## Experiment 1

As noted, individuals often conform in the absence of any apparent social or economic gain^3–6^. It is unclear, however, whether conformity also occurs in the face of conspicuous loss. Critically, in the neuroeconomic literature, the price that a participant is willing to pay for a commodity is a common measure of its value^17–21^. In Experiment 1, the decision to conform often came at a price. Specifically, participants chose between gambling options that differed in terms of the probability of a fictitious monetary reward (henceforth the “pay-off”), given an ostensible majority endorsement by previous gamblers of the option associated with either a smaller or larger pay-off.

## Methods

### Participants

Thirty undergraduates at the University of California, Irvine (19 females, mean age = 20.70 ± 2.56) participated in the study for course credit. The sample size was determined through a post hoc power analysis of data from a pilot study, indicating that 26 subjects were required for a power of 90% given a 0.05 threshold for statistical significance (*d* = 0.67). All participants gave informed consent and the Institutional Review Board of the University of California, Irvine, approved the study. All aspects of the study conformed to the guidelines of the 2013 WMA Declaration of Helsinki.

### Task & Procedure

The study used a simple gambling task in which each of six numbered slots on a game board yielded a hypothetical $1 reward with some probability. Participants were instructed at the beginning of the study that all monetary rewards were fictitious but should be treated as real. They were further told that, while they would receive initial training on the probabilities with which different slots yielded reward, they would not be told about any monetary outcomes during the actual gambling phase. They would, however, have access to decisions made by some “previous gamblers” given the same slot options, before making their choice on each gambling trial. The group of previous gamblers was stated to have been drawn from a cohort of students participating in the study during the previous academic quarter. Thus, participants made their gambling decisions given information about the norm judgment of their peers, as well as previously acquired knowledge regarding the expected pay-off associated with each available option.

In the first phase, participants were trained to criterion on the probabilities with which each slot yielded the hypothetical $1 reward. To ensure equal sampling in this phase, each slot was highlighted on 10 consecutive trials, indicating the availability of that slot only. When the participant pressed a key to select the highlighted slot, an image of a one-dollar bill or a red cross was displayed on the surface of the slot to indicate, respectively, whether or not the $1 reward was delivered. Following 10 trials with a given slot, participants were asked to rate the probability of reward for that slot. If they did not report the probability within 0.2 of the programmed probability, they had to repeat another 10 trials on that slot. After being trained on, and rating, each individual slot, participants were asked to rate the probability of reward for all slots. If they did not rate the probability of each slot within 0.2 of its programmed probability, they were required to repeat the entire pre-training phase. At the end of the experiment, to assess retention, participants again rated the probability of reward for each slot.

**Figure 1:**
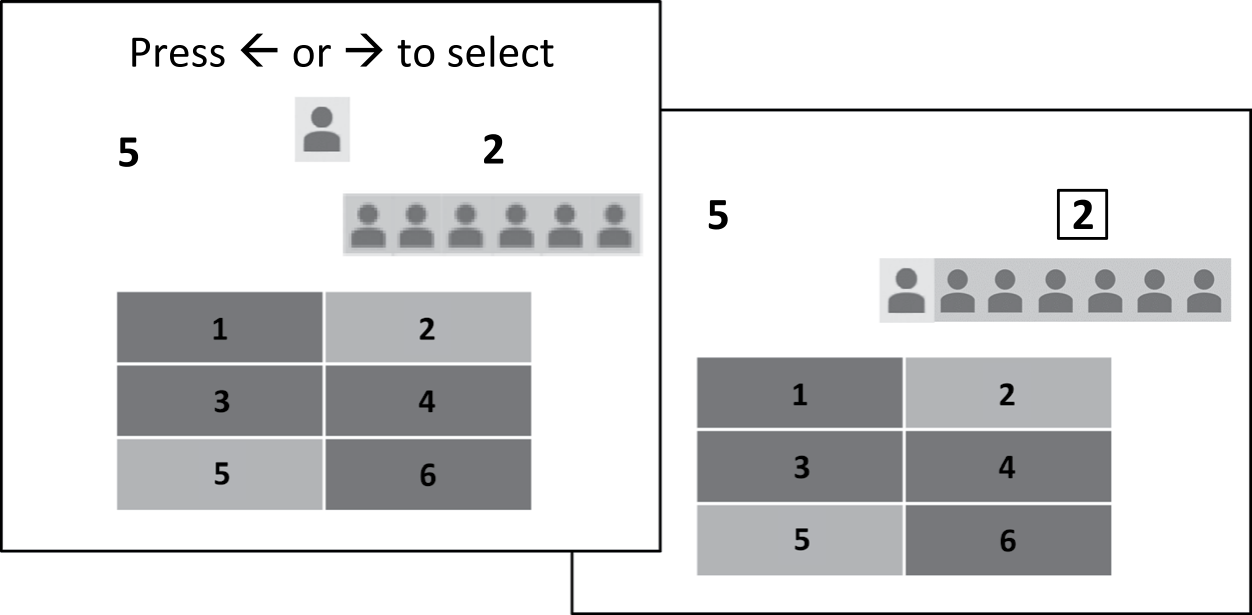
Choice and feedback screens on a trial in the gambling phase of Experiment 1. Participants pressed the left or right arrow key to indicate their choice of one of two available slot options, highlighted on the game board and displayed, respectively, to the right and left of the participant’s avatar. Following the selection, the participant’s avatar moved below the selected option, flanking a set of additional avatars that indicated the decisions of a group of previous players given the same options.

The pre-training phase was followed by a gambling phase, illustrated in Fig. 1. On each trial in the gambling phase, participants chose between two available slot options, highlighted on the board and indicated by corresponding numbers printed on the left and right side of the screen. A panel of gray icons aligned beneath available options indicated the decisions of ostensible previous gamblers given the same choice. Previous gambler icons were split across available options with a randomly determined 5–6 out of 6 majority endorsing one of the two available options. Once a participant pressed the left or right arrow key to indicate a selection, his or her personal avatar, displayed at the top center of the screen at the onset of the trial, moved below the chosen option to align with any previous gamblers already displayed beneath that option. To avoid additional learning during the gambling phase, participants were instructed that total earnings would not be revealed until the end of the experiment, but that they should assume that all outcomes were consistent with the reward probabilities established in the initial training phase.

Two of the six game board slots had a 0.2 probability of reward, two had a 0.5 probability of reward, and two had a 0.8 probability of reward. Thus, on any given trial, there was no difference, a small ($0.30) difference, or a large ($0.60) difference in expected pay-offs between the two options available on that trial. Moreover, when there was a difference, the majority of previous gamblers endorsed either the option with a lesser pay-off or that with a greater pay-off. There were 24 trials with a small difference in expected pay-offs (on 12 of these the majority of previous gamblers chose the option with a lesser pay-off and on the remaining 12 they chose the option with a greater pay-off) and 24 trials with a large difference in expected pay-offs (again, the majority chose the option with a lesser pay-off on half of those trials and that with a greater pay-off on the other half). Finally, 12 trials were included in which the expected monetary pay-off was the same for both options, yielding a total of 60 trials. The order of trials was random, with the constraints that neither a majority endorsed option nor a greater expected value option could appear on the same side of the screen on more than 3 consecutive trials, and that a particular trial type could not occur on more than 3 consecutive trials.

### Debriefing

Immediately upon completing the experiment, participants were informed that the decisions made by “previous gamblers” had in fact been generated by a computer algorithm, and were given the option to withdraw their data from the study in light of this information. All participants gave written consent to having their data included in the study after learning of the deception.

### Computational model

The expected values of gambling options were formalized as the sum over the products of the probabilities and rewards of outcome states:

1 $$EV(a)=\sum _{s\text{'}}P{(a,s\text{'})}^{\ast }R(s\text{'})$$

where *a* is the selection of a particular slot option, *P(a*,*s′)* is the probability of a particular outcome state, *s′*, given *a*, and *R(s′)* is the reward of *s*′. On each trial, each available slot option was associated with four possible outcome states: receiving $1 and agreeing with the decision of previous gamblers, receiving $1 and disagreeing with the decision of previous gamblers, receiving $0 and agreeing with the decision of previous gamblers, and receiving $0 and disagreeing with the decision of previous gamblers. Since probabilities of monetary reward were trained to criterion prior to the gambling phase, and the probability of majority affiliation could be deduced from the choice screen (see Fig. 1), *P(a*, *s*′*)* was initialized to, and maintained at, the true transition function for the events being modeled.

Two variants of the expected value model were implemented. In the first, non-social, model, the reward associated with a particular outcome state, *R(s*′*)*, was simply the expected pay-off associated with that state. To model the intrinsic value of conformity, an alternative model was also specified, in which the level of group agreement associated with a state served as a surrogate reward, such that

2 $$R(s\text{'})=m(s\text{'})+{w}^{\ast }c(s\text{'})$$

where *m(s)* is the hypothetical dollar amount associated with a slot option, *c(s)* is the relative size of the majority associated with an option and *w* is a free parameter reflecting individual differences in the value of conformity. For example, if a participant shows no preference between an option for which the probability of $1 is 0.2 and *c(s)* is 1.0, versus an option for which *c(s)* is 0.0 and the probability of $1 is 0.8, then for that particular participant, *w* should roughly equal $0.6.

**Figure 2:**
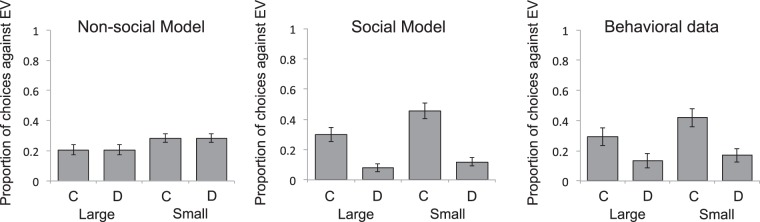
Model predictions and behavioral results from Experiment 1. Bars show mean proportions of two-alternative forced choices favoring the option with the *lower* expected monetary value (EV) in each of four conditions, defined by the magnitude of the difference in EV across available options (Large or Small) and by whether the lower EV option was associated with conformity (**C**) or dissent (**D**). In each condition, a proportion of 0.5 indicates chance performance (i.e., no preference based on EV). Subtracting the depicted proportion from 1.0 gives the proportion of choices favoring the option with a *greater* EV. The left and middle graphs respectively show mean choice probabilities generated by a non-social model of expected value and an alternative, social, model that uses majority alignment as a surrogate reward. The right graph shows participant’s actual choices. Error bars = SEM.

Both models assumed that participants select actions stochastically using probabilities generated by a softmax distribution, in which a free parameter, *τ*, controls the degree to which choices are biased toward the highest valued action. Thus, on each trial in the gambling phase, expected values were computed for the two available gambling options, using Equations 1 & 2, and the softmax rule was used to transform those values into choice probabilities, plotted, for each conformity and pay-off condition, in the left and middle panel of Fig. 2. Free parameters were fit to behavioral data by minimizing the negative log-likelihood of obtained choices for each individual using MATLAB’s fminsearchbnd function (MathWorks, 2017b), with upper-lower bounds of 0.01–1.01 for *w* (since the largest possible pay-off on a given trial was $1.00) and 0.01–100.00 for *τ*. The corrected Akaike information criterion (AICc) was used to select between models.

## Results

Statistical tests included analyses of variance (ANOVA) as well as planned comparisons, employing two-tailed t-tests or two-tailed Pearson correlations, and were calculated using *n* = 30. Effect sizes (Cohen’s *d*) and their confidence intervals are reported for all planned comparisons. Data and code are provided in the Supplementary Data.

Criterion checks at the end of the study confirmed that participants retained accurate representations of reward probabilities, with 97% of estimates falling within 0.1 of, and 92% of estimates being identical to, programmed probabilities. We also confirmed that participants were in fact incentivized by the hypothetical monetary payoffs: Collapsing across social and pay-off conditions, whenever payoffs differed across available slot options, participants chose the option with a greater payoff 75% of the time, significantly more often than chance, *p* < *0*.*0001*. Revealing a clear modulation of this preference, a two-by-two ANOVA performed on the proportion of choices favoring the slot option with a *lower* expected pay-off, with the social decision associated with that slot option (conforming or dissenting) and the size of the difference in pay-offs between options (large or small) as factors, yielded a main effect of social decision (F(29) = 7.40, *p* < 0.05, ηp² = 0.20) and a main effect of the difference in pay-offs (F(29) = 17.88, *p* < 0.001, ηp² = 0.38), as well as a marginally significant interaction (*p* = 0.06). As can be seen in Fig. 2, a comparison of model-derived choice probabilities with participants’ actual choices suggests that the model that treats social alignment as a surrogate reward dramatically outperformed the non-social model. A statistical comparison confirmed this apparent difference; mean AICc scores were significantly smaller, indicating a better fit, for the social model than for the non-social model (t(29) = 4.07, *p* < 0.001, *d* = 0.60, 95% CI [0.08, 1.12]). The means and standard deviations of the best-fitting parameters, and of the associated negative log likelihoods, are listed in Table 1.

**Table 1:** Best fitting parameter values for the learning rate (*α*), the value of conforming (*w*) and softmax noise (*τ*), together with negative log (NL) likelihoods, for the social and non-social model of expected value in Experiments 1 & 2.

	*α*	*w*	*τ*	NL Likelihood
Experiment 1				
Social	—	0.39 ± 0.38	7.33 ± 13.10	22.73 ± 9.99
Non-social	—	—	6.85 ± 17.92	30.20 ± 11.21
Experiment 2				
Social	0.23 ± 0.35	0.49 ± 0.44	20.93 ± 28.76	42.70 ± 17.42
Non-social	0.40 ± 0.40	—	8.98 ± 19.74	57.55 ± 6.42

Specifically, as predicted only by the social model, and illustrated by the means in Fig. 2, whenever the probability of reward differed across available options, participants were significantly more likely to chose the option associated with a *lower* pay-off if that option was endorsed by a majority of ostensible previous gamblers, whether the difference in pay-offs across options was large t(29) = 2.06, *p* < 0.05, *d* = 0.53, 95% CI [0.02, 1.05]) or small t(29) = 3.13, *p* < 0.005, *d* = 0.85, 95% CI [0.33, 1.38]). In other words, participants appeared willing to relinquish an alternative incentive in order to conform to the group norm.

An important consideration when interpreting these results is the fact that monetary rewards were hypothetical: It is possible, therefore, that the apparent willingness to pay a price for majority affiliation reflected a lack of awareness of, or failure to be incentivized by, monetary pay-offs. We consider this unlikely for a couple of reasons. First, because a large number of behavioral and neuroimaging studies have found similar effects of fictitious and real rewards^22–26^. Second, and more importantly, the results clearly demonstrated an overall preference for gambling options with *greater* pay-offs that increased significantly with the magnitude of the difference in pay-offs.

When the probabilities of reward were the same for both available options, participants on average chose the option endorsed by a majority of previous gamblers 66% of the time, significantly greater than chance; t(29) = 2.66, *p* < 0.05, *d* = 0.49, 95% CI [0.11, 0.88]. Importantly, these choice preferences did not depend on the degree to which a participant had learned or retained the pay-off probabilities: the accuracy of rated reward probabilities for all gambling slots obtained at the end of the study did not predict the degree to which participants favored options associated with conformity over dissent, *p* = 0.64, nor did it predict the difference in AICc scores between social and non-social algorithms, *p* = 0.94. Likewise, the free parameter *w*, which reflects individual differences in the value of conformity, was predicted neither by the accuracy of recalled reward probabilities at the end of the study, *p* = 0.97, nor by the number of training rounds required to learn those probabilities to criterion at the beginning of the study *p* = 0.54.

Critically, the choice preferences reported above replicate those of an exploratory study (see Supplementary Fig. S1), identical to Experiment 1 except that all decisions by ostensible previous gamblers were unanimous, and that ostensible others were simply referred to as “previous players”, rather than stated to have been drawn from a cohort of students participating in the study during the previous academic quarter. The replication provides support for the robustness of our results.

By pitting conformity against an alternative incentive, Experiment 1 provided evidence for the hypothesized value of majority alignment. But how was that value established? Previous work has demonstrated that group agreement results in greater levels of experienced group membership^1,2^, greater monetary payoffs^27^, superior memory retrieval^28^ and more accurate perceptual judgments^29^. Indeed, an aggregate of judgments by a group of individuals often outperforms that of any member of the group; a phenomenon referred to as the “wisdom of the crowd”^30^. Thus, the valence of apparently inconsequential social alignment may have been acquired through a history of contingent success and reward. While it is not practically possible to evaluate this hypothesis by tracing each individual’s reinforcement history, a corollary and tractable prediction is that, once established, such valence should in turn “rub off” on any stimulus, contextual or interpersonal, associated with majority affiliation. This prediction is tested in Experiment 2.

## Experiment 2

The ability of hedonic stimuli to transfer valence to neutral stimuli with which they are paired, termed *conditioned reinforcement*, has been studied extensively using a wide range of stimuli, species and procedures^31–34^. Once established, previously neutral conditioned reinforcers can pass on their motivational significance to other neutral stimuli: For example, casino chips maintain gambling based on their association with monetary reward, which in turn obtains valence from its usefulness in acquiring primary rewards. One might expect, therefore, that any sufficiently valuable stimulus, no matter how abstract, should be able to induce conditioned reinforcement in associated arbitrary, initially neutral, stimuli. In Experiment 2, we assess the degree to which the valence of conformity and dissent decisions are transferred to concomitant stimuli. In particular, we explore how the rewarding properties of social conformity may modulate a previously demonstrated increase in the rated likability of visual stimuli paired with monetary reward, as well as the preference for such stimuli when placed in a novel choice context^35^.

A prominent theory of consensus seeking is that it reflects an attempt to escape from the cognitive dissonance^36^ elicited by a mismatch between one’s own judgment and that of other individuals^4,15^. This account implies two things: First, if measured against a neutral baseline, valence associated with conformity and dissent should be asymmetric, since it is the negative affect associated with dissent that motivates conformity. Second, a direct experience of conformity or dissention, such that it is *one’s own* decisions that conflict with those of others, should be required for affect to emerge. An alternative perspective is that states defined by high levels of agreement or dissent may have been symmetrically associated with gains and losses respectively, acquiring positive and negative valence accordingly. Moreover, features associated with agreement or dissent may elicit affect in the absence of any directly experienced conflict as when observing conformity, or lack therefore, among other individuals. In Experiment 2, to arbitrate between these accounts, we assessed the degree to which motivational significance is attributed to ostensible other individuals engaging in conforming and dissenting decisions.

## Methods

### Participants

Thirty undergraduates at the University of California, Irvine (23 females, mean age = 20.26 ± 2.01) participated in the study for course credit. All participants gave informed consent and the Institutional Review Board of the University of California, Irvine, approved the study. All aspects of the study conformed to the guidelines of the 2013 WMA Declaration of Helsinki.

### Task & Procedure

**Figure 3:**
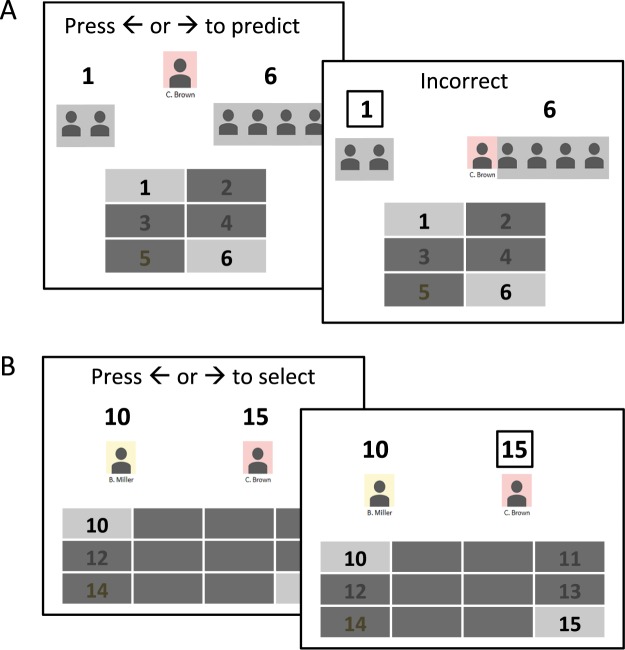
Illustration of trials in Experiment 2. (**A**) Choice and feedback screens on a trial in the social learning phase. Participants pressed the left or right arrow key to predict which option the target gambler would chose. On the subsequent screen, the participant’s selection was indicated by a square around the chosen option, the target gambler’s avatar moved below the option ostensibly selected by the target gambler, flanking a set of additional avatars that indicated the decisions of several non-descript players given the same options, and accuracy feedback was displayed center-screen. *(***B**) Choice and feedback screens on a trial in the gambling phase. Participants pressed the left or right arrow key to select one of two slot options drawn from a set of novel options with unknown reward probabilities. On critical trials, a conforming and dissenting target gambler from the previous phase respectively endorsed each slot option. On the feedback screen, the participant’s selection was indicated by a square around the chosen option.

As in Experiment 1, at the start of the study, participants were instructed that they would be playing a game in which they would be required to select between pairs of slots on a game board, with each slot yielding a fictitious monetary reward ($1) with some probability. Following initial training to criterion on the reward probabilities (identical to Experiment 1), participants were instructed that, before playing the game themselves, they would have an opportunity to learn more about the game by observing the choices of previous gamblers (as in Exp. 1, previous gamblers were stated to have been drawn from a cohort of students participating in the study during the previous academic quarter). On each trial in this “social learning” phase, participants were shown the game board with two highlighted available slots, the choices made by several previous gamblers (indicated, as in Experiment 1, by gray icons aligned below numbers corresponding to available slots), as well as a distinctly colored and named target gambler displayed at the top center of the screen (see Fig. 3A). The assignment of colors and name-tags to target gamblers was randomized across subjects.

Participants were asked to predict the choices of the target gambler on each trial; following this prediction, a selection square appeared around the slot indicated by the participant while the target gambler moved below the slot number they had ostensibly selected on that trial (either the same or opposite of that predicted by the participant), aligning with any non-specific gamblers already displayed beneath that option. The choices of non-specific previous gamblers (i.e., the panel of gray icons beneath a relevant option) were split across available options with a randomly determined 4–6 out of 6 majority. As in Experiment 1, there was either a zero, small ($0.30) or large ($0.60) difference in expected pay-offs between available slot options on each trial, with the majority of non-specific gamblers selecting the option with a greater pay-off on half of the trials and that with a lesser pay-off on the remaining trials. Critically, three target gamblers were in agreement with the majority of other gamblers on all trials, while the remaining three target gamblers dissented on all trials. Thus, participants were able to predict the decisions of target gamblers based on the distribution of non-specific previous gamblers across available options.

Finally, participants were instructed that, while no monetary outcomes of gambling decisions would be shown in the social learning phase, they should assume that the outcomes of the target gamblers’ choices reflected the reward probabilities established in the initial training phase. The number of trials with each possible pair of slot options was the same for conforming and dissenting target gamblers and is listed in Table 2. In each of nine blocks of 6 trials, both conforming and dissenting target gamblers chose slot options that yielded monetary reward with high (0.8), medium (0.5) and low (0.2) probabilities. Additional trials in which both conforming and dissenting gamblers always chose the option with a low reward probability were presented in 3 separate blocks, each with six trials, intermittently distributed throughout the sequence of blocks. As a result of these contingencies, by the end of the social learning phase, one target gambler from each social condition (conformity and dissent) was respectively associated with a cumulative reward of $5.9, $4.7, and $3.5.

**Table 2:** Possible pairings of slot reward probabilities into two-alternative forced choice scenarios during the social learning phase of Experiment 2, with the number of instances each scenario was presented to target gambler associated with high ($5.9), medium ($4.7) and low ($3.5) cumulative gain. The first number in a pair (e.g., 0.8 in 0.8–0.5) is the reward probability of the slot selected by the target gambler and the second number that of the alternative slot available on the trial.

	0.8–0.8	0.8–0.5	0.8–0.2	0.5–0.8	0.5–0.5	0.5–0.2	0.2–0.8	0.2–0.5	0.2–0.2
$5.9	1	4	1	1	0	0	1	1	1
$4.7	0	1	1	2	1	2	1	1	1
$3.5	0	0	1	0	1	2	1	4	1

Following the social learning phase, participants were asked to rate the likability of the six target gamblers on a scale from 0 (not at all likable) to 10 (extremely likable). They were then instructed that they would have an opportunity to gamble themselves, but to do so on a novel set of slots. Specifically, as illustrated in Fig. 3B, the game board used in the social learning phase was now flanked by three additional slots on each side, for a total of six new slots, thus creating a novel choice context. On each trial, two target gamblers from the social learning phase were shown respectively beneath the two available slot numbers, indicating their endorsement of that particular option.

There were 15 possible combinations of target gamblers, with each combination being repeated 8 times for a total of 120 block randomized trials. On critical trials, one available option was endorsed by a “conforming” target gambler and the other option by a “dissenting” target gambler, with the cumulative pay-off from the social learning phase being greater for either the conforming or the dissenting target gambler. Participants pressed the left or right arrow key to select between slots, for the instructed purpose of maximizing reward. They were informed that all monetary earnings would be revealed at the end of the study.

As in Experiment 1, following the gambling phase, participants again rated the probability of reward for each slot option, as well as the degree to which each target gambler had agreed with the majority of previous gamblers in the social learning phase, on a scale from 0 (never) to 10 (always). All participants were debriefed upon completing the study, using the same debriefing protocol as in Experiment 1.

### Computational model

Changes in the valence of target gamblers were formalized by an error-driven algorithm^37,38^ that incrementally updated the value of a target gambler proportional to the difference between observed and expected values on each trial. Specifically, on a given trial, the change in the value of a particular target gambler, Δ*V(s)*, was defined as:

3 $${\rm{\Delta }}V(s)=\alpha [R(s\text{'})-V(s)]$$

where *α* is a learning rate parameter and *s* represents the identifying color and name tag of a particular target gambler. *R*(*s*′*)* was defined, as in Experiment 1, for a conventional, non-social, model as well as a social model that treats conformity as a reward surrogate.

Recall that, on each trial in the “social learning” phase, a particular target gambler was paired with a particular slot outcome and with either conformity or dissent from a group majority. Critically, we assume that each slot option has acquired a value proportional to its reward probability during the initial pre-training phase (while not explicitly modeled, this transfer of valence is fully accounted for by the algorithm specified in Equation 3). We further assume that, while participants did not directly experience trial outcomes during the “social learning” phase, they learned through observation to associate target gamblers with the values of those trial outcomes (see Cooper *et al*.^39^ for a similar model of vicarious learning). Thus, on each trial in the “social learning” phase, the value of the target gambler present on that trial was updated with an amount proportional to the difference between the initial value of that target gambler and the reward probability (and conformity level) of the slot outcome on that trial (i.e., the prediction error specified on the right hand side of Equation 3). In the gambling phase, Equation 1 was again used to generate expected decision values, with *s*′ being defined as the target gambler associated with a particular slot option, and with the value, *V(s*′*)*, of that target gambler, acquired during social learning, replacing *R(s*′*)*. Note that, while participants made a prediction on each trial in the social learning phase about which slot option a particular target gambler would select, the underlying affective learning addressed by Equation 3 is instead the incremental acquisition of value by target gamblers based on their repeated pairing with high vs. low slot reward probabilities, and with conformity vs. dissent.

**Figure 4:**
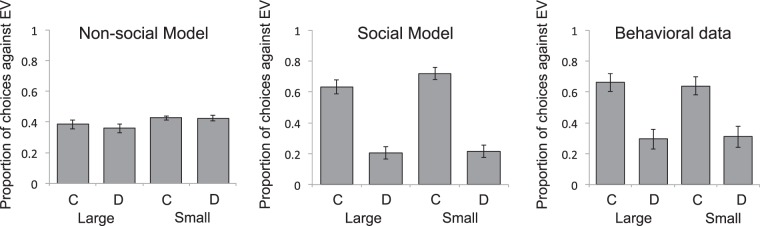
Model predictions and behavioral results from Experiment 2. Bars show mean proportions of two-alternative forced choices favoring the option endorsed by a target gambler with a *lower* cumulative monetary gain (expected value, EV, on y-axis), in each of four conditions, defined by the magnitude of the difference in cumulative gain across target gamblers (Large or Small) and by whether the gambler associated with a lower cumulative gain was associated with conformity (**C**) or dissent (**D**). In each condition, a proportion of 0.5 indicates chance performance (i.e., no preference based on the endorsing target gambler’s cumulative gain). Subtracting the depicted proportion from 1.0 gives the proportion of choices favoring the option endorsed by a target gambler with a *greater* cumulative gain. The left and middle graphs respectively show mean choice probabilities generated by a non-social model of expected value and an alternative, social, model that uses majority alignment as a surrogate reward. The right graph shows participant’s actual choices. Error bars = SEM.

As in Experiment 1, a softmax rule was use to generate choice probabilities (plotted in Fig. 4), and free parameters were fit to data by minimizing the negative log-likelihood of choices made in the final gambling phase, as participants selected between slot options endorsed by different target gamblers. Thus, the greater the value of *w*, the greater the value acquired by a conforming target gambler in the social learning phase, and the greater the probability of choosing a slot option endorsed by that gambler in the subsequent gambling phase. Fits were performed using MATLAB’s fminsearchbnd function (MathWorks, 2017b), with upper-lower bounds of 0.01–0.99 for *α*, 0.01–1.01 for *w*, 0.01–100.00 for *τ*. The corrected Akaike information criterion (AICc) was used to select between models.

## Results

Statistical tests included ANOVAs as well as planned comparisons, employing two-tailed t-tests or two-tailed Pearson correlations, and were calculated using *n* = 30. Effect sizes (Cohen’s *d*) and their confidence intervals are reported for all planned comparisons. Data and code are provided in the supplementary dataset.

The mean rated likability of each target gambler, obtained following the social learning phase, is presented in Table 3, together with corresponding model-derived state values, *V(s)*. A two-by-three ANOVA performed on the likability ratings, with social decision and cumulative gain as factors, revealed a significant main effect of social decision (F(29) = 19.50, *p* < 0.001, ηp² = 0.40) but no effect of cumulative gain (*p* = 0.23) and no interaction (*p* = 0.42). Planned comparisons revealed that mean ratings deviated significantly from the neutral point (5) on the rating scale for both conforming (t(29) = 5.04, *p* < 0.0001, *d* = 0.92, 95% CI [0.50, 1.37]) and dissenting gamblers (t(29) = 2.92, *p* < 0.01, *d* = 0.54, 95% CI [0.15, 0.0.93]); that is, social valence changed in both a positive and negative direction. In the final gambling phase, when target gamblers associated with the same amount of cumulative reward endorsed different slot options, participants’ preference for the option endorsed by a gambler associated with conformity over one associated with dissent was significantly greater than chance; 69%, t(29) = 2.76, *p* < 0.01, *d* = 0.50, 95% CI [0.12, 0.90].

**Table 3:** Mean rated likability ratings of conforming and dissenting target gamblers in Experiment 2, at each level of cumulative gain (i.e., expected value; EV), together with corresponding mean state values, *V(s)*, derived by social and non-social computational models.

	High EV	Mid EV	Low EV
Rated Likability			
Conforming Player	6.77 ± 2.13	6.90 ± 1.88	6.17 ± 2.18
Dissenting Player	3.87 ± 2.37	3.80 ± 2.31	3.73 ± 2.59
Social Model			
Conforming Player	0.47 ± 0.51	0.38 ± 0.39	0.37 ± 0.40
Dissenting Player	0.27 ± 0.26	0.22 ± 0.22	0.17 ± 0.18
Non-Social Model			
Conforming Player	0.43 ± 0.31	0.30 ± 0.22	0.25 ± 0.20
Dissenting Player	0.38 ± 0.28	0.33 ± 0.23	0.25 ± 0.21

As noted, we attribute both likability ratings and choice preferences to changes in the affective valence of target gamblers respectively associated with conformity and dissent: In other words, we conjecture that the preference for gambling options endorsed by target gamblers associated with conformity reflects an increase in the motivational salience, or value, of those players. Consistent with this interpretation, choice preferences were significantly predicted by differences between conforming and dissenting target gamblers in likability ratings, *p* < 0.001.

Importantly, the above results did not reflect a failure to learn or retain the reward probabilities associated with different slot options: participants’ ratings of reward probabilities at the end of the study were highly accurate, with 97% of estimates falling within 0.1 of, and 91% of estimates being identical to, programmed probabilities. Moreover, to the extent that they existed, deviations of estimations from programmed reward probabilities did not predict individual differences in either preference for (*p* = 0.20) or likability of (*p* = 0.57) conforming gamblers. Participants were also clearly able to discriminate between conforming and dissenting target gamblers, t(29) = 8.14, p < 0.0001, *d* = 2.76, 95% CI [2.05 3.46]; however, their ability to do so predicted neither differences in the likability of conformity and dissent gamblers, p = 0.53, nor their preference for options endorsed by conformity gamblers, p = 0.38.

A two-by-two ANOVA performed on the proportion of choices favoring options endorsed by target gamblers associated with lower cumulative reward, with social decision (conforming or dissenting) and the difference between target gamblers in cumulative gain (small or large) as factors, revealed a main effect of social decision (F(29) = 9.11, *p* < 0.01, ηp² = 0.24) but no main effect of the difference in cumulative gain (*p* = 0.90), and no interaction (*p* = 0.53). Specifically, as predicted only by the computational model that treated conformity as a surrogate reward, and illustrated in Fig. 4, when the history of conformity as well as of cumulative payoffs differed across target gamblers endorsing different options in the final gambling phase, participants chose the option endorsed by the target gambler associated with a lesser payoff significantly more often if that gambler had a history of making conforming decisions, whether the difference in cumulative payoffs was small, t(29) = 2.56, *p* < 0.05, *d* = 0.91, 95% CI [0.38, 1.44]) or large, t(29) = 3.35, *p* < 0.005, *d* = 1.07, 95% CI [0.53, 1.62]. The superior performance of the social model was confirmed by planned comparisons of AICc scores, which were significantly smaller, indicating a better fit, for the social than for the non-social model (t(29) = 5.41, p < 0.0001, *d* = 1.05, 95% CI [0.67, 1.43]).

As with differences in likability ratings and overall preferences, differences in AICc scores between the non-social and social model were not predicted by differences in the acquisition or retention of slot reward probabilities, *p*′*s* > *0*.14. Moreover, differences in learning rates derived from the social model predicted neither likability ratings, *p* = 0.72, nor choice preferences, *p* = 0.63. Finally, the value of the *w* parameter was predicted neither by the accuracy of recalled reward probabilities at the end of the study, *p* = 0.56, nor by the number of training rounds required to learn those probabilities to criterion at the beginning of the study *p* = 0.83. It might be argued, however, that in spite of the relatively large differences in slot reward probabilities, differences in the cumulative gain associated with conforming and dissenting target gamblers, based on their ostensible decisions in the social learning phase, were too subtle to be discernable, or deemed relevant, to participants in the subsequent gambling phase. To address this possibility, the gambling phase included trials on which both target gamblers were equally associated with either conformity or dissent, differing solely in terms of the monetary rewards accumulated in the social learning phase. On such trials, participants showed a significant preference for the option endorsed by a gambler associated with greater cumulative monetary gain; mean preference = 0.59 ± 0.21, t(29) = 2.29, *p* < 0.05, *d* = 0.42, 95% CI [0.04, 0.80].

The likability ratings and choice preferences reported above replicate those of an exploratory study (n = 20), identical to Experiment 2 except that differences between target gamblers in cumulative monetary reward were less pronounced (i.e., $5.3, $4.7 and $4.1 for high, medium and low cumulative gain) and that ostensible others were simply referred to as “previous players”, rather than stated to have been drawn from a cohort of students participating in the study during the previous academic quarter. The choice preferences from the gambling phase of the exploratory study are presented in Supplementary Fig. 1. A similar pattern of results was also obtained in a related experiment that used a courtroom cover-story, in which participants showed greater preference for, and increased rated likeability of, courtrooms in which their judgment of a particular court-case agreed with a unanimous jury decision, over courtrooms in which their judgment had differed from that of a unanimous jury^40^. Together, these replications provide support for the robustness and generalizability of our findings.

## Discussion

In two experiments we investigated the affective properties of agreeing or disagreeing with an ostensible group majority, by pitting conformity against a non-social incentive, and by assessing the transfer of social valence to concomitant stimuli. Using computational cognitive modeling to formalize the role of conformity in value-based decision-making, we found that models that treat agreement with a group majority as a surrogate reward provide a better account of choice preferences than do conventional algorithms. In Experiment 1, when the probability of a fictitious monetary reward differed across available options, participants chose the option associated with a *lesser pay-off* significantly more often if that option was endorsed by a majority of ostensible previous gamblers. In Experiment 2, participants demonstrated a preference for options endorsed by, and reported a greater likability of, gamblers that had a history of agreeing with majority decisions over gamblers that had a history of dissent, even when dissent gamblers were associated with greater cumulative reward. Critically, these effects were not predicted by participants’ near perfect ability to accurately recall the objective reward probabilities.

An important caveat for interpreting these results is that the monetary outcomes in our studies were hypothetical. The use of fictitious money is prevalent throughout the decision sciences, with direct comparisons showing equivalence to real monetary rewards at both behavioral and neural levels^22–26^. For example, Beattie and Loomes^41^ found that using real vs. fictitious monetary incentives did not significantly alter the common ratio effect – a classical violation of economic axioms – with analogous results reported for temporal discounting, preference reversals and framing effects^23,25,42^. Note that, rather than simply assuming, based on such findings, that participants in our study were incentivized by fictional monetary outcomes, we empirically demonstrated this value by showing a 75% overall preference for gambling options with a relatively greater pay-off. Thus, while we have not shown that people are willing to incur a real monetary loss to conform, we do provide evidence that they are willing to trade social alignment against a demonstrably rewarding alternative. Nonetheless, the use of hypothetical monetary pay-offs makes it difficult to generalize our results to real-world economic decisions. Further work is needed to explore the influence of real monetary incentives on the effects reported here.

While there are several possible sources of affect associated conformity and dissent decisions, including reinforcement history, cognitive dissonance^4,15,43^ and uncertainty aversion^44–47^, it is important to note that informational inferences may also shape behavior independently of valence^48–51^. Thus, in spite of demonstrating clear knowledge of reward probabilities, participants may have made inferences about the outcome of a given trial based on the number of individuals endorsing a particular option. Such inferences, potentially formalized by a Bayesian extension of expected value computations, could serve to reduce cognitive effort, since group norms were displayed on the screen while reward probabilities had to be retrieved, or to reduce uncertainty due to the stochastic nature of the task. Although we acknowledge these possible sources of conformity, we consider them unlikely for two main reasons: First, because the accuracy of rated reward probabilities predicted neither differences in conformity choices, nor conformity-based affective changes – it seems plausible that an informational influence of group norms would be related to the quality of contingency knowledge. Second, we found the same pattern of results in a similar previous study in which trial-by-trial feedback about the, at chance, accuracy of the majority opinion was provided^40^.

Another important consideration is how ostensible previous gamblers were perceived by participants, particularly given their often suboptimal decisions: In Experiment 1, the majority of ostensible others choose an option with a lesser expected monetary pay-off than its alternative on 40% of trials; in Experiment 2, even target gamblers with high levels of cumulative gain choose an option poorer than its alternative on 30% of trials. In contrast, participants in Experiment 1 choose a poorer option on only 20% of trials (averaged across conforming and dissenting decisions). One possibility is that participants attributed the performance of ostensible others to their level of expertise. Notably, while participants were told that they needed to rate slot reward probabilities with a high level of accuracy in order to proceed with the task, they were not informed of the accuracy criterion, nor of the actual probabilities, allowing for the inference that poor decision-makers, while able to make it through the task, had an inferior understanding of the reward structure. Further work is needed to explore how the optimality of other’s decisions influences their perceived expertise, as well as inferences about their degree of conformity.

Of course, factors relevant to informational conformity, such as majority size and expertise, are also likely to shape hedonic aspects of social alignment. For example, in Equation 2, we assume that the influence of majority size on value is linear. However, previous work assessing the relationship between majority size and conformity has been equivocal, with some observing a curvilinear influence (e.g.^52^), others reporting a linear relationship^53^ and yet others identifying a diminishing function with “each additional majority member producing a smaller increment in conformity”^54^. In Experiment 1, the majority varied only from 5 to 6 (out of 6) group members, which is not enough variability to assess linearity. Nonetheless, it is important to consider how the size of a majority opinion might shape the reinforcement signals associated with conformity. Certainly, differences in both the size and expertise of a group may have shaped past reward contingencies and, as such, come to differentially predict the value of conforming. For example, agreement with a small minority of experts may have a richer history of reward than agreement with a large majority of lay people. Formally, these issues might be addressed by replacing *c(s)* in Equation 2 with a power function, the exponent of which characterizes the shape of the relationship between majority size, or expertise, and value.

Our work contributes to a growing literature on social cognition and reward learning. It should be noted, however, that a formal application of reward-related algorithms to social conformity is often lacking in such studies. For example, while Klucharev *et al*.^4^ interpreted neural responses to disagreeing with a group norm as a reinforcement learning signal, they did not do any cognitive modeling, nor did they assess any form of reward learning or reward-based behavior. Others have focused on the application of reinforcement learning algorithms to vicarious learning, particularly with respect to the representation of self- vs. other-referenced reinforcement signals (e.g.^55^). Some, however, have addressed questions more closely related to those investigated here, using economic decision-making tasks in which participants select between options given trial-specific information about the choices, outcomes and even confidence levels of other individuals^51,56^. A critical difference between these studies and ours is that, while we trained participants to criterion on slot reward probabilities, they provided participants with little or no information about the true outcome distributions of available options. In the absence of such information, the decisions of others become a viable substitute, and the cost of conforming is unknown. In contrast, our study systematically pits the desire to confirm against an alternative incentive.

Another novel aspect of the current findings is the transfer of social valence to concomitant stimuli, demonstrated by the likability ratings and choice preferences in Experiment 2. Notably, while accounts of social conformity based on cognitive dissonance or uncertainty aversion focus exclusively on negative affect associated with dissent, likeability ratings obtained in Experiment 2 suggest that such affective changes are also driven by a positive valence associated with social alignment. From a reward learning perspective, just as the act of dissenting from a group majority may acquire negative valence through a history of being paired with dissonance or uncertainty, the act of conformity should likewise acquire positive valence through its association with reinforcing consequences, such as social approval, access to group resources, and superior perceptual and economic decisions^1,2,27–29,31,32^. Thus, a reward-based perspective accounts for the development of social valence in both directions, as well as for the further transfer of that valence to neutral stimuli that coincide with conforming and dissenting decisions, demonstrated here. It should be noted, however, that likeability ratings and gambling decisions in Experiment 2 were based on the behavior of ostensible other individuals, and that much stronger negative affect might have emerged if participants’ own decisions had been contrasted with that of the group majority. Indeed, in our previous study, in which participants’ judgments about courtroom cases directly agreed or disagreed with a unanimous jury, we found a significant likability shift only in the negative direction^40^. Further work is needed to determine the symmetry of valences associated with conformity and dissent.

Consistent with the notion that majority affiliation serves as a positive reinforcement signal, several neuroimaging studies have found greater activity in the ventral striatum (VS), a region known for encoding a reward prediction error^18,57,58^, when individuals make judgments that agree with those of a group norm relative to judgments that disagree^4,6^. However, in the absence of baseline measures, it is difficult to discern the directionality of such VS responses to social feedback, and indeed, Klucharev *et al*.^4^ interpreted the effect as a *deactivation* of the VS in response to the aversiveness of diverging from the group norm. While Klucharev *et al*.’s perspective is supported by studies showing decreased VS activity in response to aversive stimuli^59–61^, others have found that the VS is bivalent, with activity increasing in response to both appetitive and aversive stimuli^62–65^, or even nonvalent, with activity increasing to neutral but surprising stimuli^11,12^. Nevertheless, greater VS activity in response to agreement than to dissent is broadly consistent with the notion of a positive reward prediction error, elicited by the hedonic properties of social conformity.

Interpretation of VS signals is further complicated, however, by the bi-directionality of dissent employed by the relevant studies: a group’s rating of a stimulus’ subjective value (e.g., the attractiveness of a face or desirability of a food) may be either greater or lesser than a participant’s rating. While both types of deviation have been shown to deactivate the VS as group norms are revealed, Zaki *et al*.^9^ found that, during subsequent re-exposure to rated stimuli, activity in the VS, as well as in the medial orbitofrontal cortex, scaled with the *signed* difference between the participant’s rating and the group norm (similar results have been observed by others, in the ventromedial prefrontal cortex^6^ as well as the VS^8^). Such signed signals, which were consistent with the direction of changes in behavioral ratings, could reflect a retrieval of the previously experienced divergence from the group norm, or new stimulus values that had been error-adjusted towards the group reference. They are not consistent, however, with a reinforcement signal encoding the hedonic valence of majority alignment, which should simply increase in response to stimuli paired with the positive hedonics of conforming decisions and decrease in response to stimuli associated with the aversiveness of dissent. Notably, conventional demonstrations of reinforcement learning in the VS primarily entail increased activity in response to unexpected reward and, critically, the transfer of such responses to stimuli associated with reward – that is, an increased signal in response to a stimulus that is repeatedly paired with a rewarding outcome (e.g.^58^) – consistent with the affective changes demonstrated in Experiment 2. Of course, no strong inferences can be drawn about the nature of VS signaling based on our purely behavioral studies. Further neuroscientific work is needed to determine how the current results relate to the neural bases of social conformity.

In conclusion, we have used conventional measures of subjective value to explore the affective properties of conforming and dissenting decisions. Our results suggest a common value-scale for social and non-social currencies, and an ability of conforming decisions to imbue concomitant stimuli with affective significance. These findings expand on a conventional characterization of majority alignment as being either normative or informational, and contribute to a growing literature on the integration of social and motivational processes.

## Supplementary information

Supplementary Information

Dataset 1

## References

[1] Bond R, Smith PB (1996). Culture and conformity: A meta-analysis of studies using Asch’s (1952b, 1956) line judgment task. Psychol. Bull..

[2] Reysen S, Branscombe NR (2008). Belief in collective emotions as conforming to the group. Soc. Influence.

[3] Sherif M (1935). A study of some social factors in perception. Arch. Psychol. (Columbia University).

[4] Klucharev V, Hytönen K, Rijpkema M, Smidts A, Fernández G (2009). Reinforcement learning signal predicts social conformity. Neuron.

[5] Corriveau KH, Fusaro M, Harris PL (2009). Going with the flow: Preschoolers prefer nondissenters as informants. Psychol. Sci..

[6] Nook EC, Zaki J (2015). Social norms shift behavioural and neural responses to foods. J. Cognitive Neurosci..

[7] Sun S, Yu R (2016). Social conformity persists at least one day in 6-year-old children. Sci. Rep..

[8] Campbell-Meiklejohn DK, Bach DR, Roepstorff A, Dolan RJ, Frith CD (2010). How the opinion of others affects our valuation of objects. Curr. Biol..

[9] Zaki J, Schirmer J, Mitchell JP (2011). Social influence modulates the neural computation of value. Psychol. Sci..

[10] Yu R, Sun S (2013). To conform or not to conform: spontaneous conformity diminishes the sensitivity to monetary outcomes. PloS One.

[11] Horvitz JC (2000). Mesolimbocortical and nigrostriatal dopamine responses to salient non-reward events. Neuroscience.

[12] Zink CF, Pagnoni G, Martin ME, Dhamala M, Berns GS (2003). Human striatal response to salient nonrewarding stimuli. J. Neurosci..

[13] Jensen J (2007). Separate brain regions code for salience vs. valence during reward prediction in humans. Hum. Brain Mapp..

[14] Cooper JC, Knutson B (2008). Valence and salience contribute to nucleus accumbens activation. Neuroimage.

[15] Matz DC, Wood W (2005). Cognitive dissonance in groups: the consequences of disagreement. J. Pers. Soc. Psychol..

[16] Gavrilets S, Richerson PJ (2017). Collective action and the evolution of social norm internalization. P. Nat. A. Sci..

[17] Plassmann H, O’Doherty J, Rangel A (2007). Orbitofrontal cortex encodes willingness to pay in everyday economic transactions. J. Neurosci..

[18] Hare TA, O’Doherty J, Camerer CF, Schultz W, Rangel A (2008). Dissociating the Role of the Orbitofrontal Cortex and the Striatum in the Computation of Goal Values and Prediction Errors. J. Neurosci..

[19] Chib VS, Rangel A, Shimojo S, O’Doherty JP (2009). Evidence for a common representation of decision values for dissimilar goods in human ventromedial prefrontal cortex. J Neurosci..

[20] Peters J, Büchel C (2010). Neural representations of subjective reward value. Behav. Brain Res..

[21] Khaw MW, Grab DA, Livermore MA, Vossler CA, Glimcher PW (2015). The measurement of subjective value and its relation to contingent valuation and environmental public goods. PloS One.

[22] Smith VL, Walker JM (1993). Monetary rewards and decision cost in experimental economics. Econ. Inq..

[23] Kühberger A, Schulte-Mecklenbeck M, Perner J (2002). Framing decisions: Hypothetical and real. Organ. Behav. Hum. Dec..

[24] Johnson MW, Bickel WK (2002). Within‐subject comparison of real and hypothetical money rewards in delay discounting. J. Exp. Anal. Behav..

[25] Madden GJ, Begotka AM, Raiff BR, Kastern LL (2003). Delay discounting of real and hypothetical rewards. Exp. Clin. Psychopharm..

[26] Kang MJ, Rangel A, Camus M, Camerer CF (2011). Hypothetical and real choice differentially activate common valuation areas. J. Neurosci..

[27] Toyokawa W, Kim HR, Kameda T (2014). Human collective intelligence under dual exploration-exploitation dilemmas. PloS One.

[28] Harris CB, Barnier AJ, Sutton J (2012). Consensus collaboration enhances group and individual recall accuracy. Q. J. Exp. Psychol..

[29] Gürçay B, Mellers BA, Baron J (2015). The power of social influence on estimation accuracy. J. Behav. Decis. Making.

[30] Lee MD, Zhang S, Shi J (2011). The wisdom of the crowd playing The Price Is Right. Mem. Cogn..

[31] Arroyo M, Markou A, Robbins TW, Everitt BJ (1998). Acquisition, maintenance and reinstatement of intravenous cocaine self-administration under a second-order schedule of reinforcement in rats: effects of conditioned cues and continuous access to cocaine. Psychopharmacology.

[32] Goldberg SR (1973). Comparable behavior maintained under fixed-ratio and second-order schedules of food presentation, cocaine injection or d-amphetamine injection in the squirrel monkey. J. Pharmacol. Exp. Ther..

[33] Katz JL (1979). A comparison of responding maintained under second-order schedules of intramuscular cocaine injection or food presentation in squirrel monkeys. J. Exp. Anal. Behav..

[34] Williams BA (1994). Conditioned reinforcement: Neglected or outmoded explanatory construct?. Psychon. B. Rev..

[35] Cox SM, Andrade A, Johnsrude IS (2005). Learning to like: a role for human orbitofrontal cortex in conditioned reward. The J. Neurosci..

[36] Festinger, L. *A Theory of Cognitive Dissonance*. (Stanford University Press, 1957).

[37] Rescorla, R. A. & Wagner, A. R. A theory of Pavlovian conditioning: Variations in the effectiveness of reinforcement and nonreinforcement in *Classical Conditioning II: Current* Research *and Theory* (eds. Black, A. H. & Prokasy, W. F.) 64–99 (Appleton-Century-Crofts 1972).

[38] Sutton, R. S. & Barto, A. G. *Reinforcement Learning: An Introduction*. (MIT press 1998).

[39] Cooper JC, Dunne S, Furey T, O’Doherty JP (2012). Human dorsal striatum encodes prediction errors during observational learning of instrumental actions. J. Cognitive Neurosci..

[40] Mistry, P. & Liljeholm, M. The Intrinsic Cost of Dissent. In *Proceedings of the 40th Annual Meeting of the Cognitive Science Society*, Kalish, C., Rau, M., Zhu, J. & Rogers, T. T. editors, number 40 in All CogSci Conferences, 786–791 (Cognitive Science Society, Austin, 2018).

[41] Beattie J, Loomes G (1997). The impact of incentives upon risky choice experiments. J. Risk Uncertainty.

[42] Grether DM, Plott CR (1979). Economic theory of choice and the preference reversal phenomenon. Am. Econ. Rev..

[43] Klucharev V, Munneke MA, Smidts A, Fernández G (2011). Downregulation of the posterior medial frontal cortex prevents social conformity. J. Neurosci..

[44] Sherif M, Harvey OJ (1952). A study in ego functioning: Elimination of stable anchorages in individual and group situations. Sociometry.

[45] McGarty C, Turner JC, Oakes PJ, Haslam SA (1993). The creation of uncertainty in the influence process: The roles of stimulus information and disagreement with similar others. Eur. J. Soc. Psychol..

[46] Smith JR, Hogg MA, Martin R, Terry DJ (2007). Uncertainty and the influence of group norms in the attitude–behaviour relationship. Brit. J. Soc. Psychol..

[47] Petrocelli JV, Tormala ZL, Rucker DD (2007). Unpacking attitude certainty: attitude clarity and attitude correctness. J. Pers. Soc. Psychol..

[48] Deutsch M, Gerard HB (1955). A study of normative and informational social influences upon individual judgment. J. Abnorm. Soc. Psych..

[49] Raafat RM, Chater N, Frith C (2009). Herding in humans. Trends Cogn. Sci..

[50] Toelch U, Bach DR, Dolan RJ (2013). The neural underpinnings of an optimal exploitation of social information under uncertainty. Soc. Cogn. Affect. Neur..

[51] Campbell-Meiklejohn D, Simonsen A, Frith CD, Daw ND (2016). Independent neural computation of value from other people’s confidence. J. Neurosci..

[52] Rosenberg LA (1961). Group size, prior experience, and conformity. J. Abnorm. Soc. Psych..

[53] Gerard HB, Wilhelmy RA, Conolley RS (1968). Conformity and group size. J. Pers. Soc. Psychol..

[54] Latané B, Wolf S (1981). The social impact of majorities and minorities. Psychol. Rev..

[55] Joiner J, Piva M, Turrin C, Chang SW (2017). Social learning through prediction error in the brain. NPJ Sci. Learn..

[56] Tomlin D, Nedic A, Prentice DA, Holmes P, Cohen JD (2013). The Neural Substrates of Social Influence on Decision Making. PloS One.

[57] McClure SM, Berns GS, Montague PR (2003). Temporal prediction errors in a passive learning task activate human striatum. Neuron.

[58] O’Doherty JP, Dayan P, Friston K, Critchley H, Dolan RJ (2003). Temporal difference models and reward-related learning in the human brain. Neuron.

[59] Maeda H, Mogenson GJ (1982). Effects of peripheral stimulation on the activity of neurons in the ventral tegmental area, substantia nigra and midbrain reticular formation of rats. Brain Res. Bull..

[60] Besson C, Louilot A (1995). Asymmetrical involvement of mesolimbic dopaminergic neurons in affective perception. Neuroscience.

[61] Ungless MA, Magill PJ, Bolam JP (2004). Uniform inhibition of dopamine neurons in the ventral tegmental area by aversive stimuli. Science.

[62] Jensen J (2003). Direct activation of the ventral striatum in anticipation of aversive stimuli. Neuron.

[63] O’Doherty JP, Buchanan TW, Seymour B, Dolan RJ (2006). Predictive neural coding of reward preference involves dissociable responses in human ventral midbrain and ventral striatum. Neuron.

[64] Seymour B, Daw N, Dayan P, Singer T, Dolan R (2007). Differential encoding of losses and gains in the human striatum. J. Neurosci..

[65] Levita L (2009). The bivalent side of the nucleus accumbens. Neuroimage.

