# Supplementary material for: The Expression and Transfer of Valence Associated with Social Conformity
Source: Sci Rep. 2019 Feb 15;9:2154. doi: 10.1038/s41598-019-38560-4 (PMC6377616; doi:10.1038/s41598-019-38560-4)
Supplement: Supplementary file 1 — Supplementary Information [file 41598_2019_38560_MOESM1_ESM.pdf]

The Expression and Transfer of Valence Associated with Social Conformity

Prachi Mistry[1] and Mimi Liljeholm[1]*

[1]Department of Cognitive Sciences, University of California, Irvine

*Corresponding author:

Mimi Liljeholm

Department of Cognitive Sciences

2312 Social and Behavioral Sciences Gateway

University of California

Irvine, CA, 92697-5100

E-mail: m.liljeholm@uci.edu

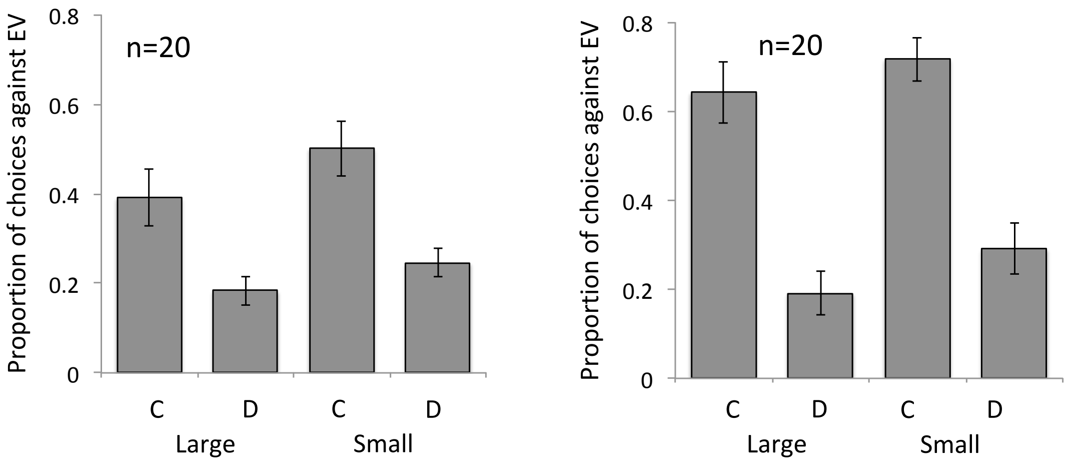

Supplementary Figure 1. Behavioral results from two exploratory studies corresponding to Experiment 1 (left) and Experiment 2 (right) respectively. Bars show mean proportions of two-alternative forced choices favoring the slot option with the lesser expected monetary value (left), or that endorsed by a target gambler with a lesser cumulative monetary gain (right), in each of four conditions, defined by the magnitude of the difference in monetary value (Small or Large) and by whether an option is associated with conformity (C) or dissent (D). See main text for details. Error bars=SEM.
