# Supplementary material for: The Expression and Transfer of Valence Associated with Social Conformity
Source: Sci Rep. 2019 Feb 15;9:2154. doi: 10.1038/s41598-019-38560-4 (PMC6377616; doi:10.1038/s41598-019-38560-4)
Supplement: Supplementary file 2 — Dataset 1 [file 41598_2019_38560_MOESM2_ESM.zip › Supplementary Dataset/Title page.pdf]

The Expression and Transfer of Valence Associated with Social Conformity

Prachi Mistry[1] and Mimi Liljeholm[1]*

[1]Department of Cognitive Sciences, University of California, Irvine

*Corresponding author:

Mimi Liljeholm

Department of Cognitive Sciences

2312 Social and Behavioral Sciences Gateway

University of California

Irvine, CA, 92697-5100

E-mail: m.liljeholm@uci.edu
